# Pelvic Malunion: A Systematic Review, Dichotomy of Definitions and Treatment

**DOI:** 10.3390/medicina58081098

**Published:** 2022-08-14

**Authors:** Sasha Stine, Austen Washington, Ramesh Kumar Sen, Kerellos Nasr, Rahul Vaidya

**Affiliations:** 1 Detroit Medical Center; 3990 John R Street, Harper 7-Brush, Detroit, MI 48201, USA; 2 Max Hospital Mohali, Phase 6 Mohali (PB), Chandigarh 160055, India

**Keywords:** pelvis 1, malunion 2, pelvic ring 3, pelvic trauma 4, pelvic fracture 5

## Abstract

*Background and Objectives*: Pelvic nonunion and malunion have been documented as rare complications in pelvic fractures and literature describing these topics is severely limited. Articles dedicated solely to pelvic malunion are nearly nonexistent. We conducted a literature search with the goal of providing a summary of the definition, causes, treatment strategies, and outcomes of pelvic malunion correction. *Materials and Methods*: An initial review of the literature was performed using the PubMed, ScienceDirect, and Cochrane Database of Systematic Reviews databases. Search terms used were “malunion” AND “pelvic” OR “pelvis”. Duplicate articles, non-English language articles without translations available and non-human subject studies were excluded. *Results*: Eleven original publications were found describing experiences with pelvic malunion. Seven of the articles were exclusively dedicated to the topic of pelvic fracture malunion, and only two reported on a series of patients treated for malunion with variably staged procedures. Most reports define pelvic pain as the main indication for surgical correction, along with gait disturbance, standing or sitting imbalance, and urinary or sexual dysfunction. Radiographically, vertical displacement of one to two centimeters and rotation of the hemipelvis of fifteen degrees or more have been described in defining malunion. No treatment algorithms exist, and each patient is treated with a unique work-up and operative plan due to the complexity of the problem. Only one series reported a patient satisfaction rate of 75% following malunion treatment. Conclusions: Pelvic malunion is a rare complication of pelvic ring injury and is seldom discussed in the literature. We found two small case series reporting exclusively on malunion treatment and complications. While some of the combination studies made the distinction in the diagnosis of malunion and nonunion, they rarely differentiated the treatment outcomes between the two categories. This paper describes pelvic malunion and highlights the need for more research into surgical outcomes of treatment specifically regarding functionality, patient satisfaction, and recurrence of preoperative symptoms.

## 1. Introduction

Pelvic ring fractures are commonly caused by high-energy trauma, typically motor-vehicle collisions and falls from a height [1,2,3,4]. In high-energy traumatic events, the affected individual commonly presents with multiple injuries involving the musculoskeletal system and other organ systems. In such circumstances, life-threatening visceral and central nervous system pathology commonly takes precedence in the immediate post-injury period. Historically, pelvic fractures commonly received initial nonoperative treatment including bed rest, postural reduction, closed manipulation, slings, casting techniques, and turnbuckles accompanied by external fixation [2,4,5,6]. However, as resuscitation protocols and fracture stabilization techniques evolved, an increasing number of patients who sustain pelvic ring injuries go on to survive them [7,8,9]. Pelvic malunion is a rare complication that occurs when fractures are inappropriately assessed and treated resulting in deformities that have a detrimental physiological impact on the patient. Current literature describing pelvic malunion is limited and as such prompted further research. This paper seeks to explain the cause of pelvic malunion; symptoms and potential deformities associated with pelvic malunion; the indications for treatment as well as the type of surgical procedures commonly performed; and the success rate of surgical correction of pelvic malunion, including reports on patient satisfaction.

## 2. Search Methods

We performed a systematics review of online scientific databases to find articles describing pelvic malunions, treatment algorithms or case reports. PubMed, Cochrane library and ScienceDirect were searched for articles on malunion with the keywords “malunion” AND “pelvic” OR “pelvis” to be included in the title. One hundred and seven articles were initially found dating between 1981 and 2022. Duplicate articles were excluded. Articles describing treatment of non-human subjects were excluded based on their title or upon review of the abstracts. Articles were also excluded due to their being published in non-English language journals with no available translations. Referential analysis of the remaining articles provided additional material. Articles were included according to the following criteria: (1) population included in the publication all sustained pelvic ring fractures that resulted in a malunion, (2) underwent surgical treatment for a malunion, (3) outcomes and/or patient satisfaction was discussed in acceptable manner. Case reports were included in the review to include all relevant literature. Review papers were excluded.

## 3. Results

Eleven publications were found describing pelvic malunions using the methodology described.

### 3.1. Cause and Rates of Malunion

The literature reporting on pelvic malunion and its causes is sparse. Several authors attribute the cause of pelvic malunion to inappropriate initial treatment of pelvic fractures [2,6,10,11]. One review observed pelvic malunion in 80% (44 out of 55) of patients who were treated nonoperatively for their pelvic injury. The authors of another review reported malunion in 37% (57 out of 153) of patients who were nonoperatively treated with pelvic ring fractures of varying degrees of severity [12]. The same article reported the rate of malunion at 43% (277 patients out of 640) when reviewing those treated by external fixation [12]. Pelvic ring injuries treated with trapezoidal external fixator have also been described with a rate of malunion reported at 58% (64 out of 110 patients) [13].

### 3.2. Symptoms and Deformities

**Figure 1:**
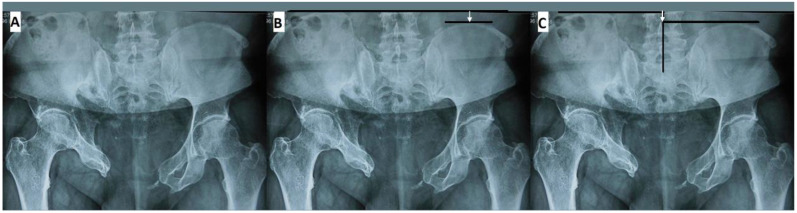
(**A**) Anteroposterior radiograph of a pelvic malunion with cranial vertical displacement of the right hemipelvis; (**B**) anteroposterior radiograph demonstrating measurement of vertical displacement of the right hemipelvis compared to the left (white arrow); (**C**) anteroposterior radiograph demonstrating measurement of vertical displacement of each hemipelvis along perpendicular vertical reference line (white arrow).

Minimal literature is available defining pelvic malunion. Clinical assessment for symptoms as well as radiographic examination are key in developing the diagnosis. Utilization of plain radiography and computed tomography (CT) can help assess the pelvic deformity and state of the osseous union. The symptoms of pelvic malunion are myriad and include sitting discomfort, standing imbalance, sexual issues, urinary and bowel dysfunction, dyspareunia, insomnia due to pelvic instability, vaginal impingement, gait disturbance, and low back pain [1,2,3,4,5,6,12]. However, severe, debilitating pain localized in the pelvic area is the most common complaint among those with pelvic malunion [3,4,14]. Low back pain complaints can be misleading as the pain can be due to previous neurologic injury [4,5]. The presence of symptoms indicates the possibility of pelvic malunion; nevertheless, radiographic investigation is necessary for diagnosis. There is no widely accepted definition of pelvic malunion. However, fracture fixation principles dictate that restoring length, alignment, and rotation restores function. Vertical displacement can be measured by drawing lines from the highest point of the ilium, perpendicular to the corresponding lumbar vertebra or sacrum (Figure 1) [4]. Henderson also described a method of measuring vertical displacement in which a straight vertical central line is drawn through the lower lumbar/upper sacral area, horizontal lines perpendicular to the center line is drawn across the superior-most point of each iliac wing and the distance between these two horizontal lines is the displacement (Figure 1) [5].

**Figure 2:**
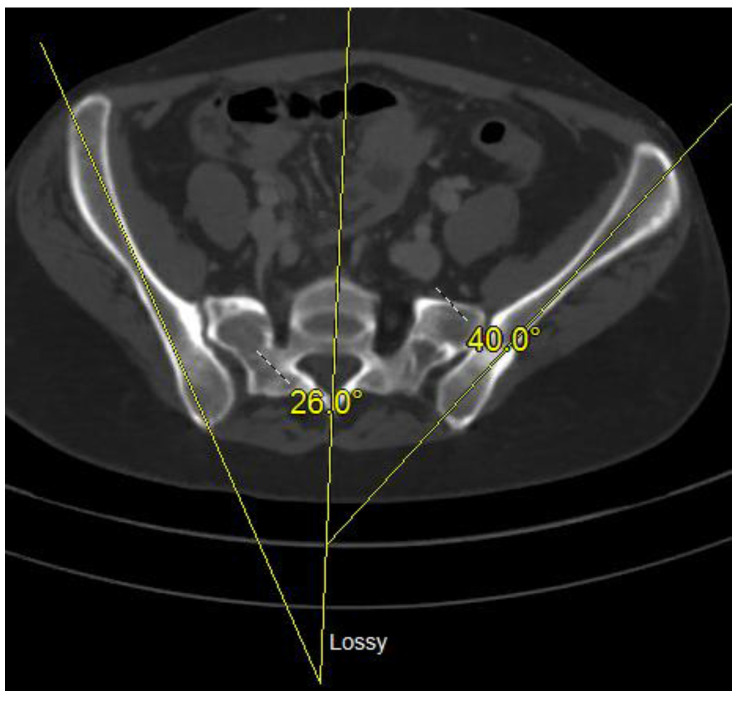
Axial CT image of a pelvic malunion with internal rotation deformity of the right hemipelvis.

**Figure 3:**
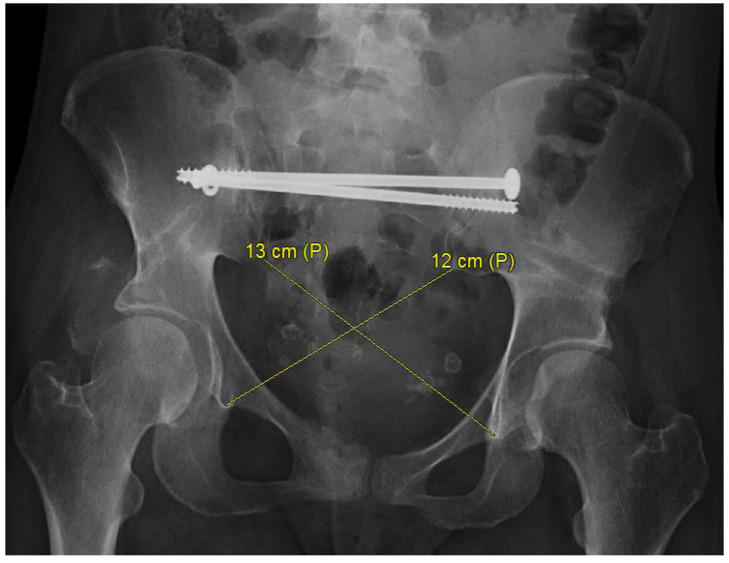
Anteroposterior radiograph of a pelvic malunion with internal rotation deformity of the right hemipelvis demonstrating diagonal distance from the low border of the sacroiliac joint to the bottom of the radiographic tear drop in skeletally mature patients.

Symphysis disruption can be measured on anteroposterior radiograph of a pelvis as the distance between the vertical aspects of the pubis bones. Anterior to posterior displacement can be measured on an inlet radiograph as the distance between two horizontal reference line drawn along the posterior aspect of each iliac crest [4]. Rotational deformity can be measured on an axial image of a pelvis by drawing a line through the middle of the sacrum and measuring the angles from this line to the lines drawn along each iliac bone (Figure 2) [4]. Rotational deformity and the quality of reduction can also be assessed by using the Keshishyan Deformity Index [15]. Two diagonal lines are drawn from sacroiliac joint to contralateral triradiate cartilage, as described in the original pediatric article. Alternatively, radiographic tear drop can be used (Figure 3). The difference between the two diagonal lines should not be more than four millimeters, and that difference divided by the sum of diagonal measurements defines the deformity index. The quality of reduction can be judged on percent improvement of the Keshishyan Index (KI) between preoperative and postoperative anteroposterior pelvis radiographs. 

A dichotomy exists between acceptable initial fracture reduction compared to malunion definition. Anatomic fracture reduction at the time of injury is deemed necessary. However, clinically relevant malunion definitions vary. Malunion and nonunion have been described as a spectrum of post-traumatic deformity [16]. Cano-Luis et al. defined malunion as pelvic ring deformity that is stabilized and nonunion as deformity that is unstable. Based on the radiographic assessment, malunion has been observed in patients with one or more of the following parameters: vertical displacement or leg length discrepancy of more than one centimeter, internal or external malrotation of the hemipelvis of more than 15 degrees, anterior-to-posterior hemipelvis displacement of more than one centimeter, and difference in Keshishyan diagonal lines of more than four millimeters [2,10,12,13,17].

### 3.3. Indication for Treatment and Surgical Techniques

Chronic pain, cosmetic asymptomatic disfigurement, pelvic instability, and deformities that affect physiologic function are the main indicators for surgical intervention [2,6,17,18,19]. Corrective surgery for pelvic malunion is technically difficult, time consuming, and accompanied with substantial blood loss; thus, extensive preoperative planning is necessary to determine the surgical approach and appropriate osteotomy technique for the associated deformity [2,6,10,13,19,20]. Various indications, results, and outcomes have been reported (Table 1). Preoperative radiographic analysis most commonly involves anteroposterior, inlet, and outlet plain radiographs as well as a standard pelvic CT scan and 3D CT scan to locate and define the deformity [2,3,4]. Recently, it has been suggested that dynamic stress examination under anesthesia and fluoroscopy can provide a more accurate assessment, diagnosis, and treatment of pelvic ring deformities [21,22]. With the advancement of technology, recent preoperative planning has included virtual 3D modeling for more precise visualization of pelvic deformities. Kurz et al. input images and data from CT scans of the pelvis into finite element modeling software and were able to view and manipulate the virtual pelvis in a near exact image of the actual pelvis. This allowed for the modeling of the instrumentation placement at the exact scale of the pelvis upon which the surgical procedure would take place [10]. Operative treatment can be extensive with average time of approximately six hours [2,6]. Oransky and Tortora reported a mean blood loss of 700 mL (range of 200 mL to 5000 mL) with average operative time of six hours (range of two to ten hours) [2]. Similar statistics were reported in a systematic review of pelvic malunion treatment: 106 patients with average blood loss of 1194 mL (ranging 12 mL to 7200 mL) and average surgical time of 6.14 h (ranging 1.1 to 10.4 h) [6]. Surgical intervention, done in a single operation or staged fashion, varied with the type and number of approaches used along with osteotomies depending on the severity of the malunion and the type of associated deformity [14,17]. The three stage method is the most routinely used in pelvic malunion and deformity correction with the posterior or anterior approach dependent upon the deformity [2,3,4,6,18]. One report suggested delayed osteosynthesis after initial malunion takedown and applied skeletal traction in cases with limb length discrepancy of more than three centimeters [16]. Progressive skeletal traction used to correct the limb length discrepancy over a seven-to-ten-day period after the initial operation is used to decrease neurovascular injury. Foregoing the correction of the original deformity and restoring limb length with a transiliac osteotomy has also been described [19] . Transiliac osteotomy usually reserved for cases in which correcting the deformity at the injury site would put neurovascular structures at unacceptable risk. Intraoperative monitoring using somatosensory evoked potential (SSEP) was utilized in several reported cases to minimize the risk of neurologic deficit due to injury of the surrounding pelvic nerves [4,18,23]. Single stage reconstructions aimed at addressing a specific problem, such as bladder entrapment or herniation, has also been reported [24,25].

### 3.4. Surgical Outcomes and Complications

The main treatment goals of pelvic malunion are deformity correction, pain relief and achieving union. Operative malunion deformity correction is technically demanding, and patients are at risk of intraoperative and postoperative complications. These challenging patient situations are typically approached with methodical preoperative imaging, planning and multiple operations to achieve satisfactory results [6]. Unions rates for nonunion and malunion correction have been reported between 88% and 100% (Table 1). In one large series of malalignment correction consisting of 134 patients, varying severities of malunion were reported to have achieved complete anatomical correction in 67 (50%) patients, satisfactory correction in 47 (35%) patients, and unsatisfactory alignment in 20 (15%) patients [4]; 76 (57%) patients reported to be extremely satisfied, 43 (32%) patients were satisfied, and 15 (11%) patients were unsatisfied with their surgical outcomes [4]. Another series recorded satisfactory but not anatomical deformity correction in 12 of 55 patients (21%) with treatment of malunion and nonunion, achieving less than 1 cm of vertical displacement and/or less than 15 degrees or rotational malalignment [2]. The most striking benefit reported was improved walking ability in all patients. A smaller series reported three (37.5%) patients were extremely satisfied after corrective surgery, three (37.5%) patients satisfied, and two (25%) patients were unsatisfied with the outcome of their surgical treatment [19]. However, it was reported that all eight patients had relief of their lower back pain and six (75%) patients were able to ambulate without assistive devices. Complication rates reported vary between 21% [4], 24% [2], and 75% [19]. Associated complications included implant failure, pulmonary embolism, delayed wound healing, deep wound infection, persistent pain, persistent malunion, impaired gait, nerve injuries, intraoperative bladder and vascular injuries [1,2,6,12,19]. Discrepancies between deformity correction, functional status and patient satisfaction are described in most series and possibly attributable to the extensive surgical morbidity and postoperative complications including persistent pain and disability. 

## 4. Discussion

Pelvic fractures often present with severe life-threatening traumatic injuries that require immediate intervention and can result in delayed or inappropriate imaging and treatment. Inadequate management methods including nonoperative treatment of displaced fracture patterns and/or insufficient or short-term fixation of unstable fractures are the main causes of malunion. Malunion of pelvic ring injuries create deformities, such as leg length discrepancy and rotational abnormalities that negatively affect physical function. Aside from physical disability the most reported symptom of pelvic malunion is severe disabling pain [3,4,12]. Pain and deformity recognized through plain radiographs and CT scans are the indications for surgical correction. [2,6] The purpose of pelvic malunion surgery is to restore anatomical symmetry and relieve any associated symptoms [2,6,14]. However, perfect anatomic reduction is not always achieved in malunion surgery. The dichotomy of acceptable initial fracture reduction and definition of operable malunion which requires an intervention is striking. Pelvic malunion surgery is technically challenging, time consuming, accompanied by considerable blood loss and require appropriate preoperative planning [2,6,10,18,19,20]. Only two case series in current literature present a cohort of patients treated specifically for pelvic malunion, describing symptoms, treatment, and outcomes [14,19]. Rousseau et al. presented eight patients treated with two stage procedure including sacroiliac osteotomy, however no patient satisfaction was reported [14]. Transiliac osteotomy has been introduced in recent years as an alternative approach to the traditional two and three stage methods for surgical correction of pelvic malunions [10,19]. Lu et al. reported on eight patients treated with a transiliac osteotomy which allowed for a lower mean surgical time than those mentioned in the previous sections; however, they recorded a higher mean blood loss of 2225 mL, compared to standard methods [19]. Surgical intervention of any kind puts the patient at risk for intraoperative and postoperative complications. Late-stage pelvic reconstruction has an even higher rate of complications than other orthopedic reconstructions [17,18]. Surgical results are satisfactory at best and return to preinjury functionality is currently unrealistic [3,4]. Surgery should only be attempted if the malunion is symptomatic and infringes on the patient’s physical function. The probability of complications increases due to the nature of pelvic fractures being accompanied with more life-threatening injuries [18]. It is not uncommon for a patient to undergo multiple corrective surgeries to address persistent malunion or pain symptoms [2,4]. Surgical complexity and operative risks involved can explain the vast difference in acceptable radiographic measurements at initial injury reduction compared to malunion definitions. The literature exclusively studying pelvic malunion is limited. While some of the studies made the distinction in diagnosis of malunion and nonunion, they rarely differentiated the treatment outcomes between the two categories. This review seeks to describe solely pelvic malunion and will hopefully inspire more research into pelvic malunion surgical outcomes especially functionality, patient satisfaction, and occurrence of preoperative symptoms.

## 5. Conclusions

Pelvic malunion is a panful, function-limiting, stable deformity and is a rare complication of pelvic ring injury. This is seldom discussed in the literature. We found two small case series and several case reports discussing malunion treatment and complications. The large series frequently cited for malunion definitions present nonunion and malunion patients in the same cohort. While some of the combination studies made the distinction in the diagnosis of malunion and non-union, they rarely differentiated the treatment outcomes between the two categories. The most common definition of malunion includes vertical displacement of the injured hemipelvis by one centimeter or more and internal or external malrotation of the hemipelvis by more than 15 degrees resulting in symphysis overlap or widening. Keshishyan Index is commonly used to evaluate rotational deformity correction. This paper describes pelvic malunion and highlights the need for more research into surgical outcomes of treatment specifically regarding functionality, patient satisfaction, and recurrence of preoperative symptoms.

## Figures and Tables

**Table 1:** Malunion definitions, clinical signs, reported surgical times and outcomes.

Author	Number of Malunion Patients (Total in the Series)	Radiographic Malunion Definition	Clinical Signs	Average Operative Time (hrs.)	Average Blood Loss (mL)	Number of Patient with Union (%)	Patient Satisfaction (%)
**Mears and Velyvis** [4]	70 (204)	ER/IR * > 15° Symphysis Gap/Overlap > 1 cm AP Displacement > 1 cm Vertical Displacement > 1 cm	LLD LE Malrotation	-	-	195 (96)	90
**Oransky and Tortoro** [2]	44 (55)	Vertical Displacement > 1 cm	LLD	6	700	54 (98)	-
**Matta et al.** [20]	18 (37)	No definition	LLD	7	1977	36 (97)	86
**Vanderschot et al.** [18]	1 (2)	No definition	LLD	8	2000	2 (100)	100
**Rousseau et al.** [14]	8	No definition	LLD > 2 cm	4.6	-	7 (88)	-
**Lu et al.** [19]	8	No definition		4.1	2225	8 (100)	75
**Kurz et al.** [10]	1	Mears criteria	LLD 4 cm	-	-	1 (100)	-
**Frigon and Dickson** [23]	1	IR of the hemipelvis > 15 °	IR LE Deformity	7	-	1 (100)	-
**Lee et al.** [15]	1	Hemipelvis Displacement > 1 cm Rotation 15–20°	LLD	-	-	-	-
**Herteleer et al.** [24]	1	No definition	ER LE Deformity	-	-	1 (100)	-
**Fang et al.** [25]	1 (5)	No definition		-	-	1 (100)	100

* ER—external rotation; IR—internal rotation; LLD—limb length discrepancy; LE—lower extremity.

## Data Availability

Not applicable.
